# Urinary KIM-1 as a Marker of Renal Tubular Injury Associated with Urethral Obstruction in Non-Azotemic Cats

**DOI:** 10.3390/ani16121907

**Published:** 2026-06-19

**Authors:** Francisco Antônio Félix Xavier Júnior, Steffi Lima Araujo, Thyago Habner de Souza Pereira, Tiago Lima Sampaio, Alice Maria Costa Martins, Nina Bezerra de Morais, Ana Raquel Almeida Pinheiro, Isadora Oliveira de Carvalho, Hélio Noberto de Araújo Júnior, Isaac Neto Goes da Silva, Janaina Serra Azul Monteiro Evangelista

**Affiliations:** 1 Centro de Educação, Ciências e Tecnologia da Região dos Inhamuns, Faculty of Veterinary, State University of Ceará, Tauá 63660-000, Ceará, Brazil; helio.noberto@uece.br; 2 Comparative Experimental Morphology Laboratory, Faculty of Veterinary, State University of Ceará, Fortaleza 60714-903, Ceará, Brazil; steffi.lima@uece.br (S.L.A.); glayciane.morais@uece.br (N.B.d.M.); ioc.isadora@gmail.com (I.O.d.C.); janaina.azul@uece.br (J.S.A.M.E.); 3 Animal Physiology Laboratory, Institute of Animal Health and Production, Faculty of Veterinary, Federal Rural University of the Amazon, Belém 66077-830, Pará, Brazil; thyagohabner1@gmail.com; 4 Department of Clinical and Toxicological Analysis, Faculty of Pharmacy, Dentistry and Nursing, Federal University of Ceará, Fortaleza 60430-275, Ceará, Brazil; tiagosampaio91@gmail.com (T.L.S.); martinsalice@gmail.com (A.M.C.M.); 5 Veterinary Clinical Pathology Laboratory, Faculty of Veterinary, State University of Ceará, Fortaleza 60714-903, Ceará, Brazil; mvanaraquel@gmail.com (A.R.A.P.); isaac.neto@uece.br (I.N.G.d.S.)

**Keywords:** renal injury, renal biomarkers, KIM-1

## Abstract

Cats with urethral obstruction may develop tubular damage before conventional indicators of renal dysfunction become abnormal, making early recognition challenging when relying solely on conventional renal biomarkers. This study evaluated urinary kidney injury molecule-1 (KIM-1) concentrations in non-azotemic cats with urethral obstruction and compared its expression with conventional renal biomarkers. Twenty-four male cats were prospectively enrolled and allocated into a control group (*n* = 12) and a urethral obstruction group (*n* = 12), all presenting serum creatinine concentrations within the reference interval. Urinary KIM-1 concentrations were quantified by enzyme-linked immunosorbent assay (ELISA) and normalized to urinary creatinine concentrations. Serum creatinine and blood urea nitrogen (BUN) did not differ significantly between groups. In contrast, urinary KIM-1 concentrations were significantly higher in cats with urethral obstruction compared with healthy controls (*p* < 0.001). These findings suggest that increased urinary KIM-1 expression may be associated with renal tubular injury in non-azotemic cats with urethral obstruction, even in the absence of alterations in conventional renal biomarkers. Therefore, urinary KIM-1 may represent a useful non-invasive marker of renal tubular injury associated with obstructive urinary disease. Further prospective studies incorporating additional renal biomarkers, histopathological evaluation, and longitudinal follow-up are warranted to better define its clinical applicability.

## Simple Summary

Renal tubular injury associated with urethral obstruction may occur in cats before alterations in conventional renal biomarkers become apparent. In this study, urinary concentrations of kidney injury molecule-1 (KIM-1), a biomarker of tubular epithelial injury, were evaluated in non-azotemic cats with urethral obstruction and compared with those of healthy cats. Cats with urethral obstruction presented significantly higher urinary KIM-1 concentrations despite having serum creatinine and blood urea nitrogen concentrations within reference intervals. These findings suggest that urinary KIM-1 may provide additional information regarding renal tubular injury associated with urethral obstruction and support further investigation of its clinical utility in feline medicine.

## 1. Introduction

Acute kidney injury (AKI) can be defined as a spectrum of diseases associated with a sudden onset of renal parenchyma injuries, more typically characterized by generalized kidney failure to meet the body’s excretory, metabolic and endocrine demands [1,2]. The main causes of AKI include the prolonged and indiscriminate use of nephrotoxic drugs, such as non-steroidal anti-inflammatory and chemotherapy drugs, infectious diseases, vasculitis, neoplasms, and obstruction of the urinary tract [1,3,4]. In cats, urethral obstruction is the primary cause of AKI [5].

The clinical signs of AKI can range from acute uremia to anuria that can lead to renal parenchymal injury and the development of chronic kidney disease (CKD) [1,3]. Therefore, identifying this condition in cats at early stages is crucial to prevent kidney disease progression to kidney failure. However, early diagnosis of AKI in this species is challenging due to the current limitations of diagnostic approaches in detecting subclinical kidney lesions [6].

The advent of urinary biomarkers and the classification system for AKI are important milestones in understanding the bidirectional relationship between AKI and CKD in humans [4]. Epidemiological studies have shown that even transient kidney dysfunction, such as prerenal azotemia caused by dehydration, is a risk factor for the development of CKD if the cause of azotemia is not previously corrected [7,8]. Clinical studies in humans and cats critically ill with AKI report only a slight increase in serum creatinine (sCre) concentration (0.1–0.2 mg/dL), while studies evaluating early biomarkers such as kidney injury molecule-1 (KIM-1) and galectin-3 reveal an increase in concentration before increases in sCre [9].

However, despite advances in veterinary nephrology, the early diagnosis of AKI in cats remains challenging, particularly in non-azotemic patients. Conventional renal biomarkers, such as serum creatinine and blood urea nitrogen (BUN), primarily reflect glomerular filtration function and typically increase only after substantial loss of functional renal mass [4,9]. In contrast, structural biomarkers, including kidney injury molecule-1 (KIM-1), provide direct information regarding tubular epithelial injury and may be detected before measurable changes in glomerular filtration occur [10,11,12]. Consequently, structural biomarkers have attracted increasing interest as adjunctive tools for the detection of renal injury in both human and veterinary medicine [4,10,11].

Recently, the feline Kidney Injury Molecule 1 (KIM-1) gene and its protein were described by Bland et al. [10]. KIM-1 is a tubular transmembrane glycoprotein that is not detected in the urine of patients without kidney injury [13]. The occurrence of AKI induces KIM-1 expression on the surface of undifferentiated epithelial cells of the proximal convoluted tubule. This enabled the detection of KIM-1 in the urine and tissue of cats in cases of ischemic injury, which has the potential to be used as a marker of tubular injury associated with AKI, critical disease and suspected AKI due to associated ischemic processes, including urethral obstruction [11,12].

There are few studies on the expression of early biomarkers of AKI in cats. The study by Woerde et al. [14] demonstrated increased serum galectin-3 (sGal-3) concentrations in azotemic cats with ureteral obstruction. Likewise, studies evaluating urinary KIM-1 in cats with AKI reported increased expression in cases of urinary obstruction, sepsis, and other causes of renal injury [11,14]. However, information regarding urinary KIM-1 concentrations in non-azotemic cats with naturally occurring urethral obstruction remains limited.

Most previous studies evaluating renal biomarkers have focused on established kidney injury, chronic kidney disease, or experimental models. Therefore, the present study contributes additional information regarding urinary KIM-1 expression in cats with urethral obstruction before the development of overt azotemia, improving our understanding of renal tubular injury associated with this common feline emergency. We hypothesized that non-azotemic cats with urethral obstruction would present increased urinary KIM-1 concentrations compared to healthy cats, even in the absence of alterations in conventional renal biomarkers such as serum creatinine and BUN, suggesting the presence of early renal tubular injury.

Therefore, the present study aimed to evaluate urinary KIM-1 concentrations as a marker of renal tubular injury associated with urethral obstruction in non-azotemic cats and to compare its expression with conventional renal biomarkers.

## 2. Materials and Methods

### 2.1. Study Population

Twenty-four male cats (*Felis catus*) of different ages attended at the Professor Sylvio Barroso Veterinary Hospital, State University of Ceará, Brazil, were prospectively enrolled between February 2019 and February 2021. Cats were divided into two groups: a control group (CG), composed of clinically healthy cats (*n* = 12), and a urethral obstruction group (UOG), composed of non-azotemic cats with urethral obstruction (*n* = 12).

The inclusion criteria for cats in the UOG were the presence of urethral obstruction associated with clinical signs of lower urinary tract disease, including dysuria, stranguria, pollakiuria, hematuria, and urinary house soiling, in addition to serum creatinine concentrations < 1.6 mg/dL, blood urea nitrogen (BUN) concentrations between 20 and 65 mg/dL, and urine specific gravity (USG) < 1.030.

Information regarding the duration of clinical signs associated with urethral obstruction was obtained from owner reports at admission. Although the exact duration of obstruction could not be objectively determined in all cases, owners generally reported the onset of clinical signs within approximately 24–48 h before presentation.

The diagnosis of urethral obstruction was established based on compatible clinical signs, absence of urine output, physical examination findings including a large, firm, and painful urinary bladder that could not be manually expressed, and abdominal ultrasonography demonstrating marked bladder distension. Obstruction was further confirmed during urethral catheterization and restoration of urinary flow.

All cats underwent a complete clinical evaluation at admission, including physical examination, complete blood count, serum biochemical analysis, urinalysis, quantitative urine culture, and abdominal ultrasonography. Laboratory and imaging findings were used to support the diagnosis of urethral obstruction, assess the overall clinical status of the patients, and exclude concurrent diseases that could interfere with study outcomes. Abdominal ultrasonography was performed to confirm bladder distension associated with urethral obstruction and to exclude urethrolithiasis, neoplasia, congenital urinary tract abnormalities, and ultrasonographic evidence of chronic kidney disease.

Following sample collection, cats with urethral obstruction received standard clinical management, including bladder decompression, urethral catheterization, intravenous fluid therapy, analgesia, and supportive care according to their clinical condition.

Exclusion criteria included urethrolithiasis, neoplasia, congenital urinary tract abnormalities detected by ultrasonography, urinary tract infection based on quantitative urine culture, previous or current history of lower urinary tract infection, diabetes mellitus, and hyperthyroidism. Information regarding previous lower urinary tract infections was obtained from owner reports during the admission interview and, when available, from medical records.

Cats with a history of antimicrobial or anti-inflammatory drug administration within the six months preceding admission were also excluded from the study. All urine cultures were performed prior to any therapeutic intervention, and only cats with negative urine culture results were eligible for inclusion in the study.

For the CG, clinically healthy cats presented for routine vaccination and/or elective castration were included. All healthy cats underwent a complete physical examination, and hematological, biochemical, and urinary parameters were within reference intervals for the species.

All procedures were conducted according to the ethical principles of animal experimentation approved by the Ethics Committee on the Use of Animals of the State University of Ceará (protocol 02875696/2019). All owners provided written informed consent authorizing biological sample collection, abdominal ultrasonography, and the use of clinical data for research purposes.

### 2.2. Analytical Methods

Blood samples (5 mL) were punctured from the jugular vein and separated into two aliquots: 1 mL was placed in a tube with EDTA for automatic blood count (ABX Micros ESV 60, Horiba Medical, Kyoto, Japan), and 4 mL were centrifuged for serum biochemical analysis. Serum creatinine (sCre), blood urea nitrogen (BUN), aspartate aminotransferase (AST), alanine aminotransferase (ALT), albumin and alkaline phosphatase (AF) were obtained on a biochemical analyzer (Cobas C111, Roche^®^, Basel, Switzerland) based on the enzymatic colorimetric method and spectrophotometry, following the manufacturer’s recommendations and using BioSystems^®^ (Quezon City, Philippines) reagents. Reference intervals adopted by the Clinical Pathology Laboratory of the Veterinary Hospital were 0.8–1.8 mg/dL for serum creatinine and 20–65 mg/dL for blood urea nitrogen (BUN).

All blood and urine samples from cats with urethral obstruction were collected immediately upon admission and before any therapeutic intervention, including urethral catheterization, bladder decompression, intravenous fluid therapy, or stabilization procedures. Following initial clinical evaluation and abdominal ultrasonography, urine samples were obtained by ultrasound-guided cystocentesis for laboratory analyses.

Urine samples were collected by cystocentesis, quantifying a total volume of 10 mL, and urine specific gravity (USG) was determined by refractometry (analog optical refractometer RSG32, Akso, São Leopoldo, Brazil). A conversion factor for cats was then applied using the equation: Feline USG = (0.846 × USG reading in human refractometry) + 0.154 by Reppas and Foster [15].

In addition, urine samples were analyzed using reagent strips and a semi-automatic urine analyzer (Combilizer 13, Human Diagnostics Worldwide, Wiesbaden, Germany) to obtain measurements of pH, protein, glucose, ketone, bilirubin, leukocytes, and erythrocytes in the urinalysis. Thus, an aliquot of 3 mL was centrifuged (3 min at 365× *g*), and the pellet was analyzed by light microscopy after 30 min [15].

Therefore, the measurement of urinary protein and urinary creatinine was performed with a semi-automatic biochemical analyzer (A15 BioSystems, Barcelona, Spain). The protein present in urine was determined using a commercial BioSystems^®^ kit, which is based on the pyrogallol red principle, in an endpoint reaction and reading at 620 nm. Urinary creatinine (uCre) was measured using the Jaffé alkaline picrate principle, with the commercial kit (BioSystems^®^ kit) in the endpoint reaction and reading at 505 nm. For this purpose, the urine samples were diluted 1:50 in distilled water to reach the linearity range of the test, with the final value being duly corrected. The urinary protein/creatinine ratio was the relationship between protein and creatinine values expressed in the same unit (mg/dL). Test validation was performed by determining repeatability.

All urine samples were analyzed qualitatively and quantitatively for aerobic bacteria culture, and samples with bacterial growth ≥ 105 colony-forming units/mL were considered positive [16].

### 2.3. Determination of Urinary Levels of KIM-1 by Enzyme-Linked Immunosorbent Assay

Urinary KIM-1 levels were quantified using the sandwich enzyme-linked immunosorbent assay (ELISA) (Human Kit from BosterBiological Technology, Fremont, CA, USA) for antigen quantification. Samples were measured by spectrophotometry at 450 nm, in triplicate and their values were expressed as means. A standard curve was plotted with values ranging from 12.5 to 500 pg/mL.

The detection range for KIM-1 was 12.5–500 pg/mL, and the intra-assay coefficient of variation was 3.2% expressed as a ratio to the uCre concentration. Urinary KIM-1 levels were normalized by dividing their values by the uCre values of the same sample and expressed as pg/mg of uCre (KIM-1/uCre). All measurements were performed in triplicate, and the mean value was used for statistical analysis to ensure analytical reliability.

### 2.4. Statistical Analysis

The software R (4.2.1) was used to perform all statistical tests. The means of serum creatinine, BUN, urinary density, urinary protein creatinine ratio, and KIM-1 of the CG and UOGs were compared using Student’s *t*-test. Generalized linear models (GLM) were used to test the effects of clinical status, age, weight, and castration on biochemical parameters. Firstly, the possibility of multicollinearity was excluded by calculating the Variance Inflation Factor (VIF) with the “car” package (3.1-5). All factors presented VIF < 2, therefore, none were considered problematic in the model. To evaluate the equality of variances of fixed categorical factors (clinical condition and castration), Levene’s test was used [17]. When residual diagnostics indicated non-normality of residuals, the response variable was transformed using the Boxcox function from the MASS package (7.3-65) prior to model refitting [18].

Five models were built for the following variables (response factors): serum creatinine, BUN, urinary density, urinary protein creatinine ratio, and KIM-1. In all variables, clinical status (CG × UOG), age, weight, castration, and interactions were included as fixed factors. According to Burnham and Anderson [19], these factors were removed sequentially to select the model with the lowest Akaike information criterion and correction for small sample sizes (AICs). All values were expressed as mean ± standard deviation (SD) and differences with *p*-value < 0.05 were considered significant. The individual data used for all statistical analyses are provided in the Supplementary Materials (Table S1).

## 3. Results

### 3.1. Study Population

The mean ± SD ages of cats in the CG and UOG were 2.54 ± 1.70 and 2.08 ± 1.31 years, respectively. Mean body weight was 4.45 ± 0.90 kg in the CG and 4.50 ± 0.50 kg in the UOG. No significant differences were observed between groups regarding age (t = 0.74; *p* = 0.47) or body weight (t = 0.15; *p* = 0.88).

No specific breed predisposition was identified among the evaluated cats. In the UOG, nine cats were neutered (75%) and three were intact (25%), whereas in the CG, seven cats were neutered (58.33%) and five were intact (41.67%).

Feline idiopathic cystitis associated with inflammatory urethral plugs was considered the presumptive cause of urethral obstruction in all cats included in the UOG. Obstruction was attributed to inflammatory processes associated with epithelial desquamation and urethral plug formation. Urethrolithiasis, neoplasia, congenital abnormalities, and urinary tract infection were excluded through abdominal ultrasonography, urine culture, and clinical evaluation.

All UOG cats were promptly treated, stabilized, and managed through the unblocking process. They were hospitalized and received full support to stabilize the obstructive and AKI condition, including intravenous fluid therapy, urethral catheterization, and post-procedural analgesia [methadone 0.2 mg/kg intravenously (IV) three times daily]. Urethral catheterization was performed after stabilization, asepsis, and anesthesia of the patient, with the administration of methadone 0.2 mg/kg intramuscularly (IM) with acepromazine 0.02 mg/kg (IM) initially, followed by propofol 4 mg/kg (IV), induction and maintenance with isoflurane via tracheal tube. After hospital discharge, the animals were submitted to hydrotherapy, analgesic and adjuvant therapy, according to their specific laboratory and clinical needs.

### 3.2. Measurement of Renal Markers

The data of serum and urinary renal biomarkers for CG and UOG are shown in Table 1.

**Figure 1:**
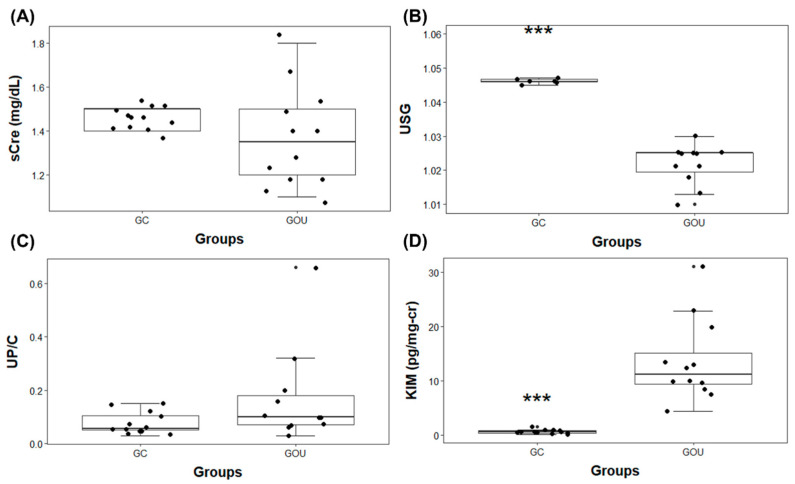
Effect of clinical status on (**A**) Serum creatinine concentration (sCre); (**B**) urine specific gravity (USG); (**C**) urine protein to creatinine ratio (UP/C) and (**D**) kidney injury molecule-1 (KIM-1) in domestic cats. Control group (CG); Urethral obstruction group (UOG) (*n* = 12, each). *** *p* < 0.05.

The sCre models did not differ from the null model, indicating that there was no effect of clinical status, age, weight, and castration on sCre levels. Furthermore, animals from the CG did not show statistical differences in relation to UOG (*p* = 0.12; Figure 1A). Similarly, the model using UP/C as predictor did not differ from the null model, with no effect of clinical status, age, weight, and castration on UP/C levels (Figure 1C).

For the model including USG as a response factor, the best model showed a significant effect of clinical status, with higher values in the CG (GLM: −0.05 ± 0.005; t = −10.09; *p* < 0.01) than in the UOG (Figure 1B). In addition, the model using KIM-1 as a response factor revealed a significant effect of clinical status, with higher expression of KIM-1 in UOG animals (GLM: 0.20 ± 0.016, t = 12.55, *p* < 0.001) than in GC animals (Figure 1D). There was no effect of weight, age, and castration in any of the models, therefore these factors were excluded from the final model.

**Figure 2:**
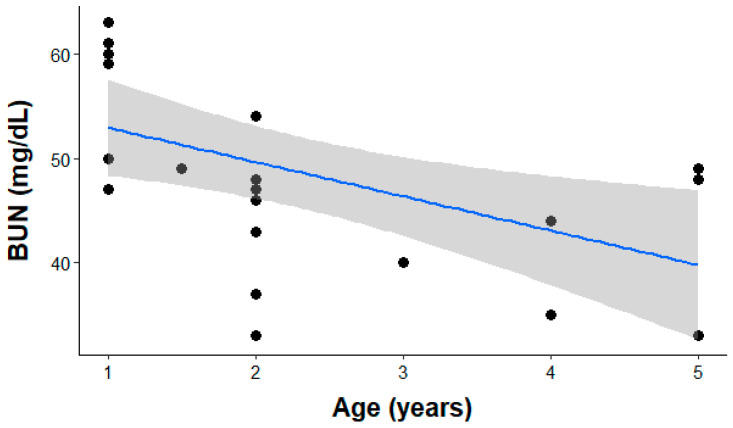
Effect of age on blood urea nitrogen (BUN) in domestic cats. (Generalized linear models—GLM: −3.28 ± 1.12; t = −2.92; *p* = 0.008). Trend line (blue line) and confidence intervals (gray band).

A last model using BUN as a response factor demonstrated a significant negative effect with age (GLM: −3.28 ± 1.12; t = −2.92; *p* = 0.008) (Figure 2). Weight, clinical status and castration had no effect in any of the models and were excluded from the final model.

## 4. Discussion

The present findings indicate that cats with urethral obstruction exhibited markedly increased urinary KIM-1 concentrations despite normal conventional renal biomarkers, suggesting an association between urinary KIM-1 excretion and tubular injury. These findings are consistent with previous studies in humans and experimental animal models of ischemia–reperfusion injury, in which KIM-1 expression increases during the early stages of renal injury [20,21].

An important aspect of the present study is that urine samples were collected before urethral catheterization, bladder decompression, fluid therapy, and other therapeutic interventions. Therefore, urinary KIM-1 concentrations reflected the clinical status of the cats at presentation, minimizing the potential influence of treatment-related factors on biomarker expression and strengthening the interpretation that increased KIM-1 levels were primarily associated with the obstructive condition itself.

Creatinine and blood urea nitrogen (BUN) are widely used markers for renal function evaluation; however, they primarily reflect glomerular filtration rather than structural renal injury. Although BUN is a sensitive marker of nitrogenous waste accumulation, its concentration may be influenced by several non-renal factors. Likewise, in cases of reduced glomerular filtration, tubular secretion of creatinine may increase, potentially masking early renal injury through an overestimation of renal function. Therefore, the need for clinically available biomarkers capable of assessing tubular health and injury is justified [4].

In the present study, neither serum creatinine nor BUN concentrations were reliable in diagnosing renal dysfunction in non-azotemic cats with urinary obstruction (UOG), with both values remaining within the reference values for the species (Table 1). We also found that creatinine was not affected by clinical status, even when controlling for age, weight, and castration. In contrast, we found that BUN was affected by age, with higher values in younger animals. This could be related to the higher protein content used in the diets of young animals [22]. Although these biomarkers are currently used to assess kidney function, sCre and BUN are considered late markers of kidney injury [9].

The lower USG observed in UOG cats may reflect impaired urinary concentrating ability associated with tubular dysfunction [15]. Furthermore, persistent hyposthenuria is suggestive of a loss of urinary concentrating capacity in the tubular segments of the nephrons [15,23]. In cats, USG ≤ 1.025 has been considered an indicator of decreased kidney function [12], and our results support this.

The inclusion criteria used in the present study were designed to select cats with urethral obstruction that remained non-azotemic according to serum creatinine concentrations, while presenting reduced urine concentrating ability. Importantly, cats were not evaluated solely on the basis of clinical history and ultrasonography. All animals underwent complete clinical and laboratory screening, including physical examination, complete blood count, serum biochemical analysis of renal and hepatic parameters, urinalysis, quantitative urine culture, and abdominal ultrasonography. Apart from findings related to the obstructive urinary condition, the remaining clinical, hematological, biochemical, urinary, and imaging parameters did not suggest systemic disease or chronic kidney disease.

Reduced urine specific gravity in cats with urethral obstruction may be physiologically explained by transient tubular dysfunction associated with increased intravesical and intrarenal pressure, altered renal perfusion, medullary washout, and impairment of tubular concentrating mechanisms [5,12,15,23,24]. Previous clinical data in cats with urethral obstruction have shown reduced urine specific gravity in obstructed cats and have also indicated that not all affected cats present overt azotemia at the time of diagnosis [12]. Therefore, the combination of serum creatinine concentrations within the reference interval and reduced USG does not necessarily indicate pre-existing chronic kidney disease. However, because SDMA, cystatin-C, histopathological evaluation, and longitudinal renal follow-up were not available, early or subclinical chronic kidney disease cannot be completely excluded. Consequently, a degree of misclassification bias remains possible and should be considered when interpreting urinary KIM-1 concentrations in cats with urethral obstruction.

Xavier Júnior et al. [12], in a clinical study evaluating dosages of KIM-1 and urinary GGT as biomarkers of acute kidney injury in cats, observed that the concentration of KIM-1 in urine is not equivalent to the concentration of conventional markers used in clinical routine; probably, this biomarker is influenced and expressed by the degree of tubular cell injury.

Previous investigations in human and experimental models have consistently reported increased KIM-1 expression following tubular injury. Some studies have also shown that KIM-1 can help differentiate ischemic AKI from prerenal azotemia and CKD and be useful in differentiating the various subtypes of AKI [25,26,27].

In the present study, we observed an increase in KIM-1 values in cats with post-renal AKI in non-azotemic patients. This increase can be explained due to the urethral obstruction, obliteration, and retention of urinary flow, which cause an increase in intravesical, ureteral, and renal pressure. This increase sustained by intrarenal pressure results in the loss of renal parenchyma and the influx of leukocytes into the renal parenchyma, releasing cytokines. As a result, there is a development of electrolyte imbalances, fibrosis in parenchyma due to the lesion, and renal ischemia secondary to dehydration caused by urethral obstruction [5]. This ischemia will lead to renal injury due to the absence of ATP, and the Na^+^/K^+^ ATPase pump will have its functioning altered, causing the entrance of calcium in tubular cells, inducing apoptosis and acute tubular necrosis [5,9,26].

Current criteria for diagnosis and classification of AKI heavily depend on sCre changes [2]. However, the timing of changes in sCre level and concomitant changes in glomerular filtration rate does not allow for an accurate estimate of time to injury or severity of dysfunction [1,8,24]. Thus, the early phase of AKI may not be associated with a significant increase in sCre levels due to several factors, including water overload, high catabolism, sepsis, and ischemic insults, resulting in delayed diagnosis and intervention. Therefore, the search for new biomarkers of AKI, especially in its initial phase, has intensified in recent years [28].

Urethral obstruction can trigger a sequence of pathophysiological events that may culminate in renal tubular injury. Urine retention increases intravesical pressure, which may be transmitted to the ureters and renal pelvis, resulting in increased intrarenal pressure and reduced renal perfusion [4,24]. This process can lead to renal ischemia, tubular epithelial cell injury, oxidative stress, inflammatory responses, and, in more severe or prolonged cases, apoptosis and tubular necrosis [4,24]. These mechanisms provide a biological rationale for the increased urinary excretion of tubular injury biomarkers, such as KIM-1, in cats with urethral obstruction [20,21,24].

The increased urinary KIM-1 concentrations observed in the present study may reflect tubular epithelial injury secondary to ischemia and increased intrarenal pressure associated with urethral obstruction. Ischemia plays a central role in the pathophysiology of obstructive renal injury, and although reperfusion is essential to restore tissue oxygenation and prevent irreversible cellular damage, it may also contribute to additional oxidative and inflammatory injury [4,24]. The reintroduction of oxygenated blood into previously ischemic tissues can intensify cellular damage through the generation of reactive oxygen species and inflammatory mediators [4,24].

Potential confounding factors should also be considered when interpreting urinary KIM-1 concentrations. Dehydration and hemodynamic alterations commonly observed in cats with urethral obstruction may contribute to reduced renal perfusion and tubular stress. In addition, all cats in the UOG presented feline idiopathic cystitis associated with inflammatory urethral plugs, and the potential contribution of lower urinary tract inflammation to urinary biomarker excretion cannot be completely excluded. Likewise, the presence of subclinical kidney disease cannot be definitively ruled out, despite the inclusion of young non-azotemic cats with no clinical or ultrasonographic evidence of chronic kidney disease. Therefore, increased urinary KIM-1 concentrations should be interpreted within the clinical context and not as an isolated indicator of renal tubular injury.

Another consideration is that urinary KIM-1 concentrations were measured while cats were still obstructed. Although urinary retention may theoretically influence urinary biomarker concentrations, KIM-1 is primarily recognized as a marker of proximal tubular epithelial injury [20,21]. Previous studies from our group demonstrated persistently increased urinary KIM-1 concentrations after relief of urethral obstruction, suggesting that elevated KIM-1 concentrations are not solely attributable to urine retention [12]. Nevertheless, the contribution of urinary retention itself cannot be completely excluded, and future longitudinal studies evaluating urinary KIM-1 before and after relief of obstruction are warranted.

This study has some limitations, including the relatively small sample size and the lack of longitudinal follow-up to assess the progression of kidney injury over time. According to IRIS guidelines, AKI diagnosis and staging are largely based on changes in serum creatinine concentration. However, non-azotemic patients with early tubular injury may not fulfill classical staging criteria despite the presence of renal damage. Therefore, cats included in the present study were interpreted as having suspected early AKI associated with urethral obstruction and evidence of early tubular injury before the onset of overt azotemia.

Another limitation of the present study is that the exact duration of urethral obstruction could not be objectively determined in all cats and was based primarily on owner-reported clinical history. Therefore, variation in the duration and severity of obstruction may have contributed to differences in the degree of tubular injury and urinary KIM-1 expression among affected cats.

Additional limitations include the absence of complementary renal biomarkers, such as SDMA and cystatin-C, as well as the lack of histopathological confirmation of tubular injury. Therefore, increased urinary KIM-1 concentrations should be interpreted cautiously as evidence of early renal tubular injury associated with urethral obstruction rather than as definitive confirmation of AKI. Further prospective studies including additional renal biomarkers, histopathological evaluation, and longitudinal follow-up are warranted to better characterize the role of KIM-1 in early feline AKI.

## 5. Conclusions

The results obtained in this study indicate that urinary KIM-1 may provide clinically relevant information regarding renal tubular injury in non-azotemic cats with urethral obstruction. Increased urinary KIM-1 concentrations were observed despite normal serum creatinine and BUN concentrations, supporting its potential application in the assessment of renal tubular injury associated with urethral obstruction. However, given the pilot nature of this study and the absence of longitudinal outcome assessment, further prospective studies incorporating additional renal biomarkers and long-term follow-up are warranted to better define the clinical role of KIM-1 in feline acute kidney injury.

## Figures and Tables

**Table 1:** Evaluation of biochemical parameters in clinically healthy cats (CG) and cats with urethral obstruction (UOG).

Parameters	CG (*n* = 12)	CI 95%	OUG (*n* = 12)	CI 95%
sCre (mg/dL)	1.46 ± 0.05	1.43–1.49	1.37 ± 0.23	1.22–1.51
BUN (mg/dL)	46.33 ± 8.86	40.71–51.96	50.92 ± 9.56	44.84–56.99
USG	1.054 ± 0.010	1.048–1.061	1.022 ± 0.006	1.018–1.026
UP/C	0.077 ± 0.042	0.050–0.104	0.170 ± 0.182	0.048–0.292
KIM-1 (pg/mg-Cr)	0.61 ± 0.38	0.37–0.85	13.54 ± 7.53	8.75–18.32

Serum creatinine (sCre), blood urea nitrogen (BUN), urine specific gravity (USG), urine protein to creatinine ratio (UP/C) and kidney injury molecule-1 (KIM-1) in domestic cats. Mean ± SD; 95% confidence interval (CI).

## Data Availability

The data presented in this study are available on request from the corresponding author.

## Supplementary Materials

The following supporting information can be downloaded at: https://www.mdpi.com/article/10.3390/ani16121907/s1, Table S1. Individual clinical and laboratory data of all cats included in the study, including age, body weight, castration status, serum creatinine (sCre), blood urea nitrogen (BUN), urine specific gravity (USG), urine protein-to-creatinine ratio (UP/C), and urinary kidney injury molecule-1 (KIM-1) concentrations used for statistical analyses.

## Abbreviations

The following abbreviations are used in this manuscript:

AKI	Acute Kidney Injury
CKD	Chronic Kidney Disease
sCre	Serum Creatinine
KIM-1	kidney injury molecule-1
CG	Control Group
UOG	Obstruction Group
BUN	Blood Urea Nitrogen
AST	Aspartate Aminotransferase
ALT	Alanine Aminotransferase
AF	Albumin and Alkaline Phosphatase
USG	Urine Specific Gravity
uCre	Urinary Creatinine
GLM	Generalized Linear Models
VIF	Variance Inflation Factor
AICs	Criterion and Correction for Small Sample Sizes
SD	Mean ± Standard Deviation
IV	Intravenously
IM	Intramuscularly
CI	Confidence Interval
UP/C	Urine Protein to Creatinine Ratio
